# Comparative biofilm-forming ability between *Clostridioides difficile* strains isolated in Latin America and the epidemic NAP1/027 strain

**DOI:** 10.3389/fcimb.2022.1033698

**Published:** 2022-12-01

**Authors:** Maria Luana Gaudencio dos Santos Morais, Mayara Gilde Castro Santos, Cecília Leite Costa, Conceição Silva Martins, Renata Ferreira de Carvalho Leitão, Dvison de Melo Pacífico, Carlos Quesada-Gómez, Débora Castelo Branco, Eliane de Oliveira Ferreira, Gerly Anne de Castro Brito

**Affiliations:** ^1^ Department of Morphology, Faculty of Medicine, Federal University of Ceará, Fortaleza, CE, Brazil; ^2^ Laboratory of Bacteriology, Department of Pathology, Faculty of Medicine, Federal University of Ceará, Fortaleza, CE, Brazil; ^3^ Laboratório de Biologia de Anaeróbios, Instituto de Microbiologia Paulo de Góes Universidade Federal do Rio de Janeiro, Rio de Janeiro, Brazil; ^4^ Facultad de Microbiología and Centro de Investigación en Enfermedades Tropicales, Universidad de Costa Rica, San José, Costa Rica

**Keywords:** *Clostridium/Clostridioides difficile*, biofilm, NaP1, toxin A (TcdA), antibiotic resistance

## Abstract

**Introduction:**

One of the challenges in treating *Clostridioides difficile* infection (CDI) is that the bacterium forms biofilms, a critical virulence mechanism known to promote antibiotic resistance and, as a result, consequently, a higher recurrence of the disease. The goal of this study was to compare the ability of three MLST Clade 2 strains to form a biofilm in vitro: ICC-45 (ribotype SLO231/UK[CE]821), a ST41 toxinotype IXb isolated in Brazil; and two epidemic NAP1/027/ST01 strains: NAP1/027/ST01 (LIBA5756), isolated during a 2010 outbreak in Costa Rica and the reference epidemic strain NAP1/027/ST01 (R20291); and ATCC700057, a non-toxigenic strain.

**Methods:**

The ability of strains to form biofilm was evaluated using crystal violet staining. In addition, samples were stained with the Film Tracer biofilm matrix (Invitrogen®) and the biofilm matrix thickness was measured using confocal microscopy. The matrix architecture was determined using Scanning electron microscop. Confocal microscopy was used to detect the presence of toxin A (tcdA) using an anti-*Clostridioides difficile* TcdA antibody. The expression of virulence genes (*tcdA*, *tcdB*, *tcdC*, *cdtB*, *spo0A*, *slpA*, *cwp66* and *cwp84*) was examined, as well as the effect of antibiotics metronidazole (MTZ) and vancomycin (VAN) on biofilm growth.

**Results:**

All of the strains tested formed a moderate biofilm with 1.1 <DO_570nm_>3.5. After 72h, biofilm biomass of the NAP1/027/ST01 epidemic strains (LIBA5756 and R20291) was significantly higher than ICC-45 and ATCC 700057 biofilms, as confirmed by electron and confocal microscopy. At 120h, the LIBA5756 biofilm biomass decreased compared to other strains. The toxigenic strains R20291 or LIBA 5756 had higher expression of genes *tcdA*, *tcdB*, *tcdC*, *cdtA*, *slpA* and *spo0A* than ICC-45, but there were no significant differences in the expression levels of *cdtB*, *cwp66* and *cwp84*. In epidemic strains, VAN and MTZ inhibited biofilm formation; however, in the ICC-45 strain, MIC concentrations of VAN and MIC and 4MIC of MTZ did not inhibit biofilm formation.

**Conclusion:**

The three MLST Clade 2 isolated from different rybotipes, two of which were isolated from Latin America, are competent biofilm-forming bacteria, indicating their ability to induce *C. difficile* infection recurrence, making treatment difficult.

## 1 Introduction

*Clostridioides difficile (C. difficile)* is an anaerobic spore-forming bacterium that is the leading cause of antibiotic-associated nosocomial diarrhea. Toxins A and B (TcdA, TcdB), binary toxin (CDT), flagella, spores and the ability to form biofilm are among the virulence factors linked to the emergence *C. difficile* infection (CDI) (Chandrasekaran and Lacy, 2017; de Melo Pacífico et al., 2021). Furthermore, prolonged hospitalization, advanced age and excessive and inappropriate use of antibiotics are risk factors for CDI (Eze et al., 2017). CDI symptoms can range from mild or severe diarrhea to serious inflammatory conditions, such as, pseudomembranous colitis, toxic megacolon, and sepsis (Czepiel et al., 2018). Some of these CDI conditions cause longer hospital stays, which has an economic impact on the healthcare system (Lessa et al., 2015; Zhang et al., 2016).

According to several studies, the hospitalization rate increased from 2001 to 2011, when it stabilized (Marra et al., 2020). The increase was primarily caused by epidemic strains (NAP1, ribotype 027), with NAP1/027 being responsible for outbreaks worldwide, including Latin American countries (Warny et al., 2005; He et al., 2013; Giancola et al., 2018; Acuña-Amador et al., 2022).

The most common circulating ribotypes in Brazil are 014, 043, 046, 106, 132, 133, 134, 135, 136, 142, 143, and 233 (Trindade et al., 2019), however, a virulent MLST Clade 2 strain, ICC-45 (ribotype SLO231/UK[CE]821), a ST41 toxinotype IXb was isolated in Northeast of Brazil from a 34-year-old female patient, diagnosed with breast cancer (Costa et al., 2016). Diarrhea began 16 days after admission and the patient died on the 54th day. Toxigenic ICC-45 (TcdA^+^, TcdB^+^ and CdtB^+^) encodes a variant of the TcdB toxin. Unlike epidemic NAP1/027 strains, strain ICC-45 is susceptible to fluoroquinolones and does not overproduce TcdA and TcdB, but it causes inflammation, oxidative stress, and epithelial damage similar to NAP1/027.

One of the most concerning aspects of CDI is its proclivity for recurrence, which occurs in 20% to 25% of patients within 4 weeks completing anti-*C. difficile* treatment (McFarland et al., 1999; Garey et al., 2008). Moreover, recurrence patients have a higher risk of death than CDI patients who do not develop a recurrence (Olsen et al., 2015). CDI recurrence may be associated with virulence factors, such as TcdA and TcdB toxin production, spore persistence and biofilm formation (Pantaléon et al., 2014; Maldarelli et al., 2016; Hamada et al., 2020).

Structured bacterial communities form biofilms that adhere to a surface, and are encased in a self-produced extracellular matrix made up of proteins, polysaccharides, DNA, and other components (Flemming et al., 2016; Karygianni et al., 2020). The extracellular matrix serves as a physical barrier to external stresses, aids in nutrient preservation, promotes antibiotic resistance, and protects against oxidative stress (Dawson et al., 2012; Dapa and Unnikrishnan, 2013). Given the importance of these biofilm characteristics, they could be valuable targets in the search for CDI treatment strategies. However, few studies on biofilm formation and characterization have been conducted. The purpose of this study was to compare the biofilms produced by two *C. difficile* isolates from Latin America to the epidemic strain NAP1/027/ST01 (R20291).

## 2 Methodology

### 2.1 Bacterial strain and culture conditions

A total of four *C. difficile* strains were studied: a toxigenic pulsotype and PCR ribotype: SLO231/UK[CE]821 (ICC45) isolated in oncology hospital in Brazil; a NAP1/ST01/RT027 strain (LIBA 5756) isolated from an outbreak in a hospital in Costa Rica; a reference epidemic strain NAP1/027/ST01 (R20291) and a non-toxigenic *C. difficile* ATCC 700057. All *C. difficile* strains were grown on Brucella agar (Oxoid^®^ - Thermo Fisher Scientific, Basingstoke, UK), supplemented with 0.1% vitamin K, 0.1% hemin (Sigma^®^, Saint Louis, USA) and 5% defibrinated sheep blood, as well as in Brain Heart infusion (BHI) broth supplemented with, 0.1% L-Cysteine, 0.1% vitamin K, 0.4% yeast extract and 0.1% hemin. The plates and tubes were incubated in anaerobic jars with an AnaeroGen™ atmosphere generation system (Oxoid^®^ - Thermo Fisher Scientific, Basingstoke, UK) at 37°C.

### 2.2 Biofilm formation assay and measurement of biofilm biomass

The protocol for the biofilm assays was adapted from Dapa and Unnikrishnan (2013). Biofilm testing was performed in 24-well polystyrene plates. In each well, an overnight *C. difficile* strain inoculum was prepared in BHI broth, diluted in a fresh BHI supplemented with 1.8% glucose (BHISG) and added. Plates were incubated under anaerobic conditions at 37°C for 24, 48, 72 and 120 h. After carefully removing the supernatant, the wells were rinsed twice with PBS and allowed to dry for 10 min. The biofilm was stained with 1 ml of 0.2% crystal violet (CV) for 30 min and incubated at 37°C. The CV was removed and the wells were washed twice with sterile PBS. One milliliter of ethanol:acetone (80:20) was used to extract the CV. The extracted dye was measured at 570nm using spectrophotometer Ultrospec 500*pro* (Amersham Biosciences). Based on the ability to form a biofilm, strains were classified into three groups, according to Pantaleíon et al., 2018 (Pantaléon et al., 2018): low-biofilm formers (OD_570_ < 1.1), moderate-biofilm formers (1.1 < OD_570_ < 3.5) and high biofilm formers (OD_570_ > 3.5).

### 2.3 Scanning electron microscopy

The *C. difficile* biofilm structure was characterized using Scanning electron microscopy (SEM), as described in Pantaleon et al., 2014 (Pantaléon et al., 2014), with some modifications. *C. difficile* strains were grown onto coverslips with a diameter of 13 mm, as described above (please see the section 2.2) in order to analyze biofilm production after 72 h. Samples were fixed in 2.5% glutaraldehyde in 0.1 M sodium cacodylate buffer for 30 min, followed by a post-fixation with 1% osmium tetroxide for 15 min. Samples were dehydrated in a series of ethanol concentrations (15%, 30%, 50%, 70%, 90% and 100%), critical point‐dried in CO_2_ and mounted on specimen stubs. Stubs were sputtered with a thin layer of gold using a Balzer’s apparatus and examined in a Quanta 450 scanning electron microscope (FEI Company), at the Central analytical at the Universidade Federal do Ceará (UFC).

### 2.4 Confocal laser scanning microscopy

Biofilm matrix was analyzed by confocal laser scanning microscopy (CLSM) according to Lázaro-Díez et al, 2016 (Lazaro-Diez et al., 2016). Biofilm assays were performed in 8-well polystyrene plates. Each *C. difficile* strain was inoculated in a fresh BHI broth supplemented with 1.8% glucose (BHISG), and 300 μL added to each well.

Plates were incubated under anaerobic conditions at 37°C for 72h. Following that, the supernatant was carefully removed, the wells were rinsed twice with PBS, and then the biofilm was stained with 200 µl per well of the FilmTracer Sypro Ruby biofilm matrix (Invitrogen), and incubated for 30 min at 37°C in the dark. The samples were examined using a LSM 510 microscope (Zeiss). The film Tracer exhibit an excitation at 488 and 561 nm and emit fluorescence at 500 nm and 550 nm, respectively. Using the LEICA software, the thickness was calculated directly from the confocal stack images.

To investigate the production of TcdA toxin, *C. difficile* strains were grown for 72h, as previously described, on 13 mm diameter coverslips. The samples were fixed in 4% paraformaldehyde in PBS at pH 7.6, washed with PBS, and permeabilized for 5 min with 0.1% Triton X-100 in PBS. Following that, the samples were then blocked for 1 h with PBS containing 5% bovine serum albumin (BSA). An anti-*C. difficile* toxin A antibody (1:300, Abcam) was added to the biofilm overnight at 4°C, followed by a secondary antibody conjugated with Alexa Fluor 596 (1:1000) for 1 h at room temperature. The images were captured using a confocal laser scanning microscope (LSM 710, Zeiss, Oberkochen, Germany). The fluorescence intensity was calculated directly from the confocal stack images using LEICA software.

### 2.5 RNA extraction and RT-qPCR analysis

The biofilm formation by *C. difficile* strains was conducted as previously mentioned (section 2.2). After 72h incubation, the supernatant was removed, and the cells were washed with cold PBS. RNA was isolated from bacterial pellets after disrupting biofilms using BHI and centrifuging the culture (14.812 g), according to the RNeasy Mini Kit (QIAGEN). The RNA concentrations were determined using the Nanodrop and cDNA was obtained using the High-Capacity cDNA Reverse Transcription kit (Applied Biosystems). The SYBR Green PCR master mix kit (Life Technologies) was used to amplify the genes tested, and the 16S rRNA gene was used as an internal control gene. The oligonucleotide primers are listed in **Table 1**. The BioRad CFX96 Real Time PCR instrument was used for the analyses

**Table 1:** Primers used in this study for real-time PCR.

Target gene	Sequence (5’-3’)	Product size	Reference
*tcdA*	5’ CAGGGCTAATAGTTTGTTTACAGAACA 3’ 5’ CAACATCTAAATATACTCCGCCAAAA 3’	143	Wroblewski et al., 2009
*tcdB*	5’ AGCAGTTGAATATAGTGGTTTAGTTAGAGTTG 3’ 5’ CATGCTTTTTTAGTTTCTGGATTGAA 3’	144	Wroblewski et al., 2009
*tcdC*	5’ GGTCATAAGTAATACCAGTATCATATCC 3’ 5’ TGAAAGACGACGAAAAGAAAGC 3’	150	Wroblewski et al., 2009
*cdtA*	5’ GATCTGGTCCTCAAGAATTTGGTT 3’ 5’ GCTTGTCCTTCCCATTTTCGATT 3’	103	Wroblewski et al., 2009
*cdtB*	5’ AAAAGCTTCAGGTTCTTTTGACAAG 3’ 5’ TGATCAGTAGAGGCATGTTCATTTG 3’	132	Wroblewski et al., 2009
*spo0A*	5’ AGCGCAATAAATCTAGGAGCA 3’ 5’AGGTTTTGGCTCAACTTGTGT 3’	117	Aldape et al., 2015
*slpA*	5’ AATGATAAAGCATTTGTAGTTGGTG 3’ 5’ TATTGGAGTAGCATCTCCATC 3’	126	Denève et al., 2009
*cwp66*	5′ GACGACGACAAGATAGTTACTCAAATTGGTGGC 3′ 5′ GACGACGACAAGATAGTTACTCAAATTGGTGGC 3’	900	Shirvan and Aitken, 2016
*cwp84*	5’ TGGGCAACTGGTGGAAAATA 3′ 5’ TAGTTGCACCTTGTGCCTCA 3’	151	Denève et al., 2009
*16S rRNA*	5’ AGCGGTGAAATGCGTAGATAT 3’ 5’ CAGCGTCAGTTACAGTCCAGA 3’	72	Aldape et al., 2015

### 2.6 Efficacy of antimicrobials against *Clostridioides difficile* biofilms

The minimum inhibitory concentrations (MIC) for metronidazole (MTZ) and Vancomycin (VAN) were determined as previously described (Costa et al., 2021). After 72 h of biofilm formation in 96-well plates, the supernatants were removed and 200μl of BHISG glucose broth containing MTZ or VAN was added to each well. CV was used to measure biofilm biomass after 48h under anaerobic incubation. Serial concentrations of MTZ (ranging from 2 to 1024 μg/mL) and VAN (from 0.125 to 64 μg/mL) were tested. For each strain, drug-free and bacterium-free wells were included as growth and sterility controls, respectively.

### 2.7 Statistical analysis

GraphPad Prism 7 software was used to perform the statistical analysis. Analysis of variance, followed by the *post hoc* Bonferroni or Tukey´s test was used to compare results that demonstrating a parametric distribution and Kruskal-Wallis’ followed by Dunn’s test for non-parametric analysis. P values of <0.05 were considered statistically significant.

## 3 Results

### 3.1 *Clostridioides difficile* biofilms

**Figure 1:**
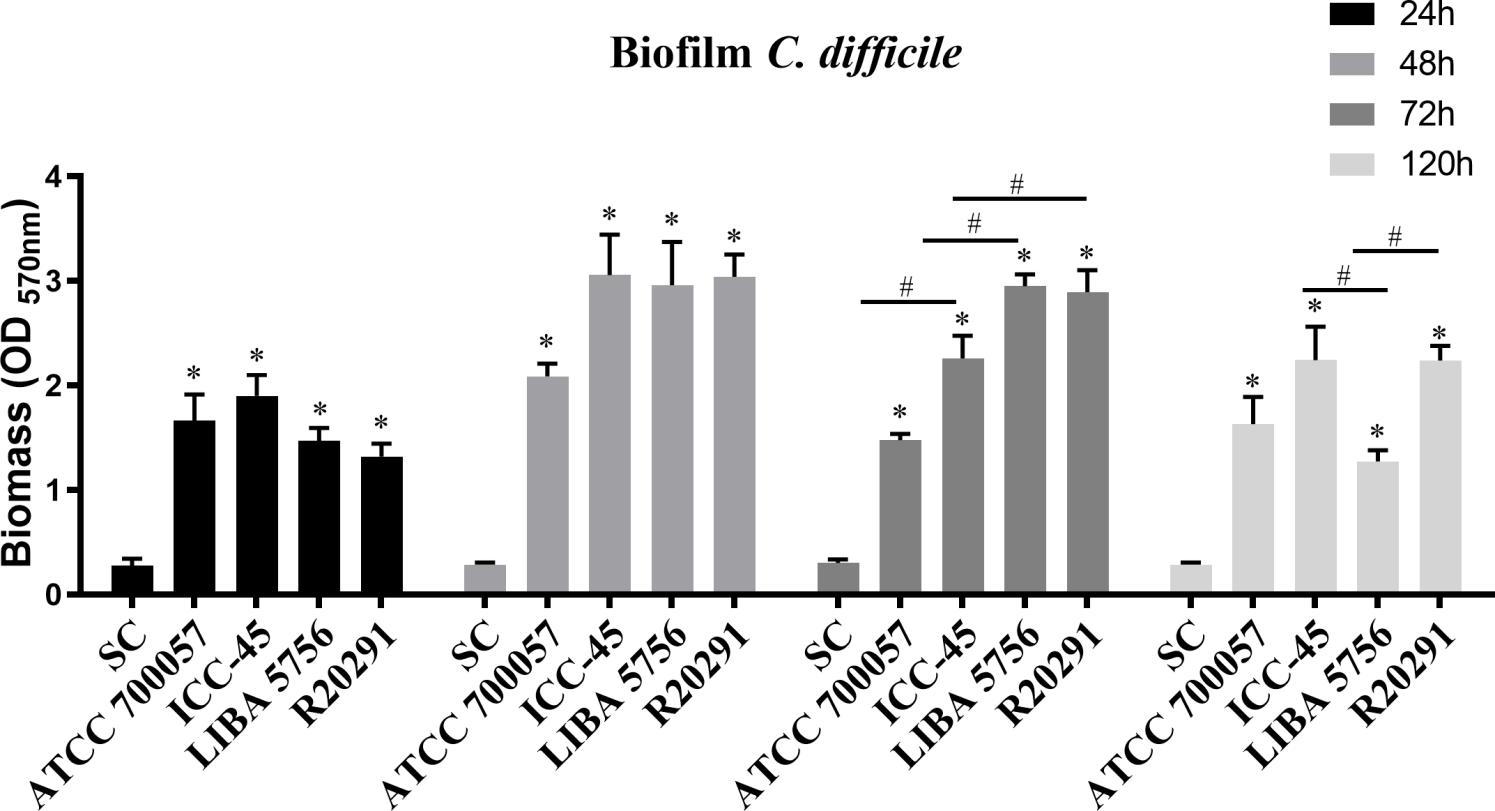
Average biofilm formation of different *Clostridium difficile* strains. Biofilm formation by strain ATCC 700057, ICC-45, and epidemic strains LIBA 5756, R20291, was measured by crystal violet staining. The biofilm formation was measured after 24h, 48h, 72h and 120 h of incubation. Error bars show standard deviations of three independent experiments, and the * represents significant differences among strains and sterility control (SC), # represents significant differences among strains. (*P* < 0.05). (One-way ANOVA followed by Bonferroni test).

The crystal violet staining assay was used to determine the biomass of biofilms. The **Table 2** shows strain category according to Pantaléon’s biofilm classification (Pantaléon et al., 2018). the strains ICC45, NAP1/027/ST01 (LIBA5756 and R20291) and the non-toxigenic strain ATCC 700057 were biofilm producers from 24h to 120h, as shown in **Figure 1**.

**Table 2:** Summary of strains and their characteristics.

Strain	Origin (geography)	Strain typing			Biofilm biomass (OD 570nm)				Classification
		MLST	PCR-ribotype	Toxinotype	Incubation time				
					24h	48h	72h	120h	
ATCC 700057	United States of America	ST48	038	Nontoxigenic	1.53	2.08	1.47	1.62	Moderate
ICC-45	Brazil	ST41	SLO231/UK[CE]821	IXb	1.83	3.05	2.25	2.3	Moderate
LIBA5756	Costa Rica	ST01	027	III	1.46	2.95	2.94	1.57	Moderate
R20291	United Kingdom	ST01	027	III	1.37	3.03	2.89	2.23	Moderate

At 48h, three clinical *C. difficile* strains formed similar biofilms, but at 72h the epidemic strains NAP1/RT027 (LIBA5756 and R20291) produced more biofilm biomass than the ICC45 and ATCC 700057 strains. However, after 120 h (**Figure 1**) of incubation, ICC-45 and R20291 showed biofilm formation persistence, with both strains having a biofilm formation capacity significantly greater than LIBA 5756.

### 3.2 ICC-45 presents a biofilm consistent with interconnected cells

**Figure 2:**
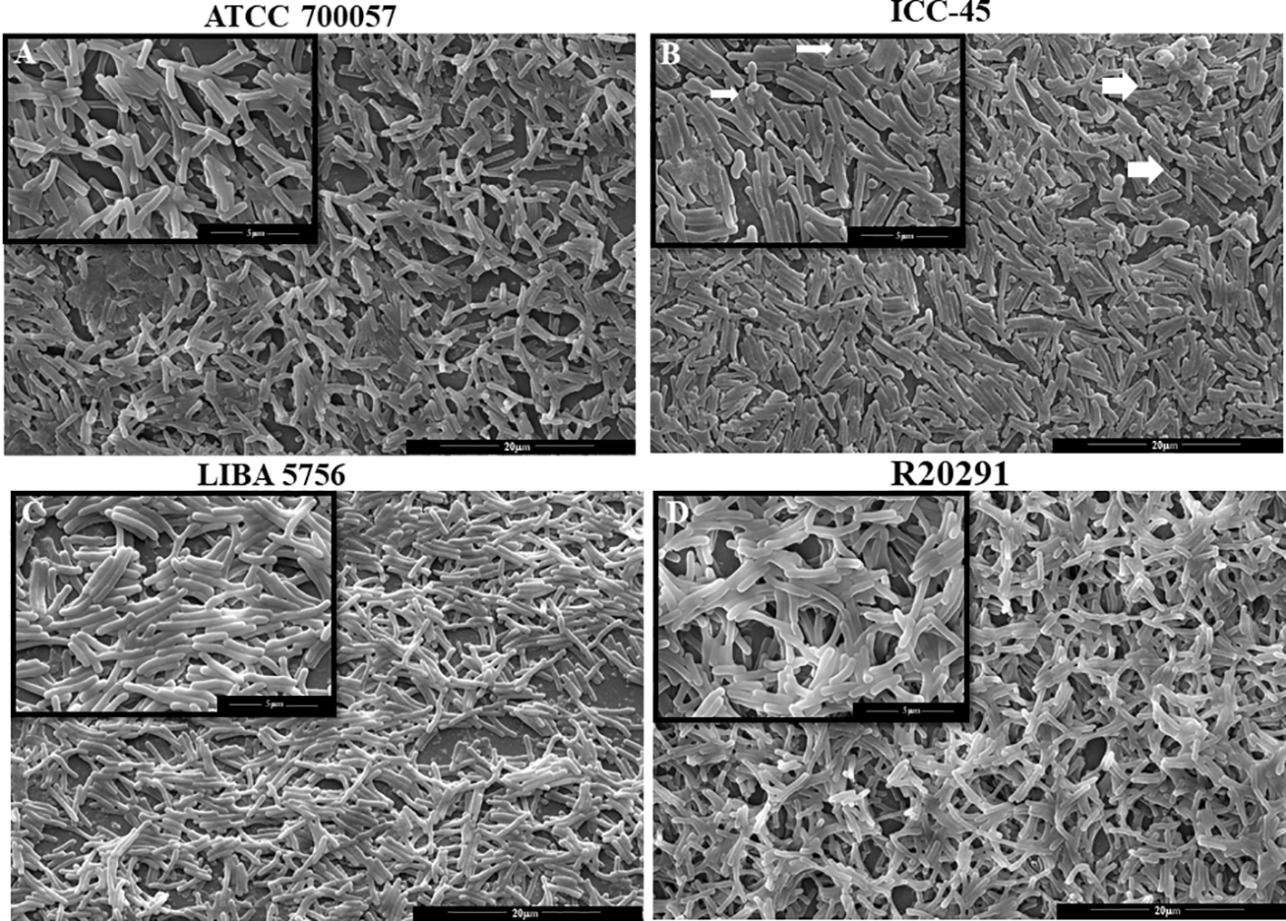
Scanning Electron microscopy (SEM) of biofilm formed by *Clostridioides difficile*. Representative SEM images of *C. difficile* biofilm architecture performed by ATCC 700057 **(A)** ICC-45 **(B)**, and epidemic strains LIBA 5756 **(C)**, R20291 **(D)** after 72h of incubation. Magnification of 5000 and 15.000 x. scale 5 and 20µm.

SEM was used to conduct a qualitative analysis of the biofilm architecture. After 72 h of incubation, SEM revealed wide mats of rod-shaped vegetative cells with interconnected cells in all strains examined. The NAP1/027 strains had a dense biofilm with masses of agglomerated cells (**Figures 2C, D**), in contrast to the ICC-45 (**Figure 2B**) and ATCC 700057 images, which had more empty spaces (**Figure 2A**). **Figure 2B** shows ovoid cells with spore-like morphological features in the ICC45 biofilm.

### 3.3 The biofilm matrix of ICC-45 was similar to the biofilm of virulent strains

**Figure 3:**
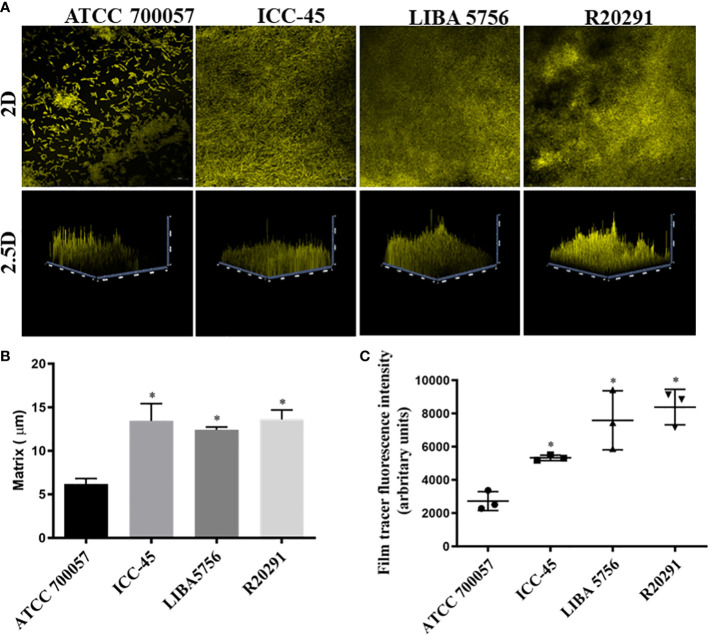
Confocal laser scanning microscopy analysis (CLSM) of 72h biofilms formed by *Clostridioides difficile*. Biofilm’s matrix formation by strain ATCC 700057, ICC-45, and epidemic strains LIBA 5756, R20291, was analyzed by FilmTracer™ SYPRO™ Ruby Biofilm Matrix Stain. **(A)** 2 D and 2.5 D Representative CLSM images of *C. difficile* biofilms **(B)** Biofilm thickness in micrometers **(C)** Mean fluorescence relative units of biofilm matrix formation * represents significant differences among strains (*P* < 0.05). Scale 20 µm. (Kruskal–Wallis test followed by Dunn’s multiple comparisons test).

The architecture of the biofilms was also examined using confocal laser scanning microscopy (CLSM). CLSM analysis revealed that clinical strain biofilms have a denser structure, thickness, and fluorescent intensity than non-toxigenic strain biofilms. **Figure 3A** shows some of the biofilms after 72 h of incubation. ATCC 700057 biofilms have a low-density matrix compared to the other strains, which is consistent with the low biofilm biomass produced at this time. Moreover, the matrix thickness of ATCC 700057 (**Figure 3B**) was significantly lower than that of ICC-45 (13.44 µm ±3.44 µm), LIBA 5756 (12.41 µm ± 0.5 µm), and R20291 (13.61 µm ± 1.8 µm). As a result, the fluorescent intensity for ATCC 700057 (**Figure 3C**) was lower than that of the other strains.

### 3.4 Toxin A production

**Figure 4:**
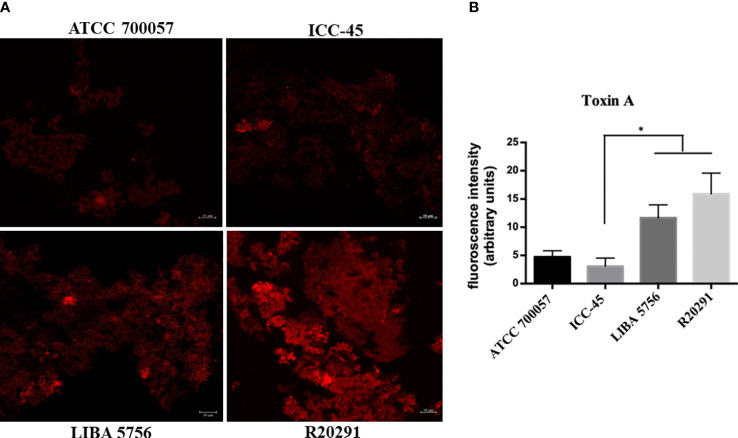
Confocal laser scanning microscopy (CLSM) of toxin A production in 72h biofilms formed by *Clostridioides difficile*. Biofilm’s TcdA production by strain ICC-45, epidemic strains LIBA 5756, R20291 and ATCC 700057 (a non-toxigenic strain) was analyzed by immunostaining with anti-*C. difficile* toxin A antibody. **(A)** Representative CLSM images of TcdA production by *C. difficile* biofilms **(B)** Mean fluorescence relative units of TcdA production by *C. difficile* biofilms. * represents significant differences among strains (P < 0.05). Scale 10 µm. (Kruskal–Wallis test followed by Dunn’s multiple comparisons test).

To determine the TcdA toxin production, the biofilms were incubated with anti-toxin A antibody and the fluorescent intensity was measured using CLSM. The ATCC 700057 and ICC-45 strains showed lower fluorescence intensities, as shown in representative images (**Figure 4A**) and in the fluorescent intensity quantification (**Figure 4B**). ICC-45 (3.07 ± 1.26) produced less TcdA than LIBA 5756 (11.65 ± 2.0) and R20291 (15.93 ± 6.32). There were no differences between LIBA 5756 and R20291 strains.

### 3.5 Expression of genes associated with virulence

**Figure 5:**
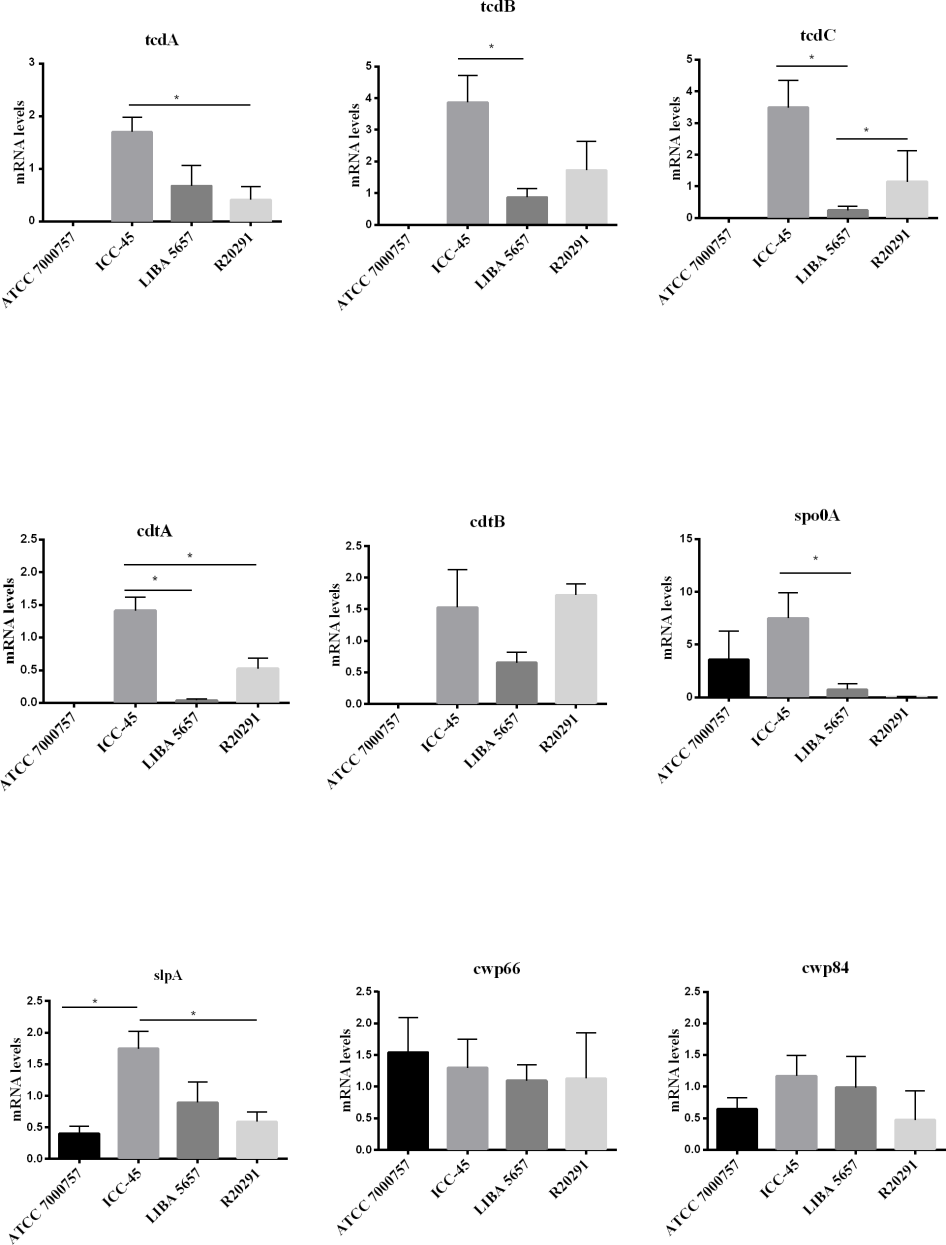
Biofilm-related gene expression of *C. difficile* strain ICC-45, epidemic strains LIBA 5756, R20291 and ATCC 700057 after 72h. * represents significant differences among strains (P < 0.05). (One-way ANOVA followed by Tukey test).

*C. difficile* virulence factors were investigated using the qRT-PCR. The ICC-45 strain expressed higher levels of *tcdA, tcdB, tcdC, cdtA*, *slpA* and *spo*0A than the toxigenic R20291 and LIBA 5756 strains. The expression levels of *cdtB*, *cwp*66 and *cwp*84 did not differ significantly between the ICC-45 and NAP1/027 strains (**Figure 5**).

### 3.6 Effects of metronidazole and vancomycin on *Clostridioides difficile* biofilms

**Figure 6:**
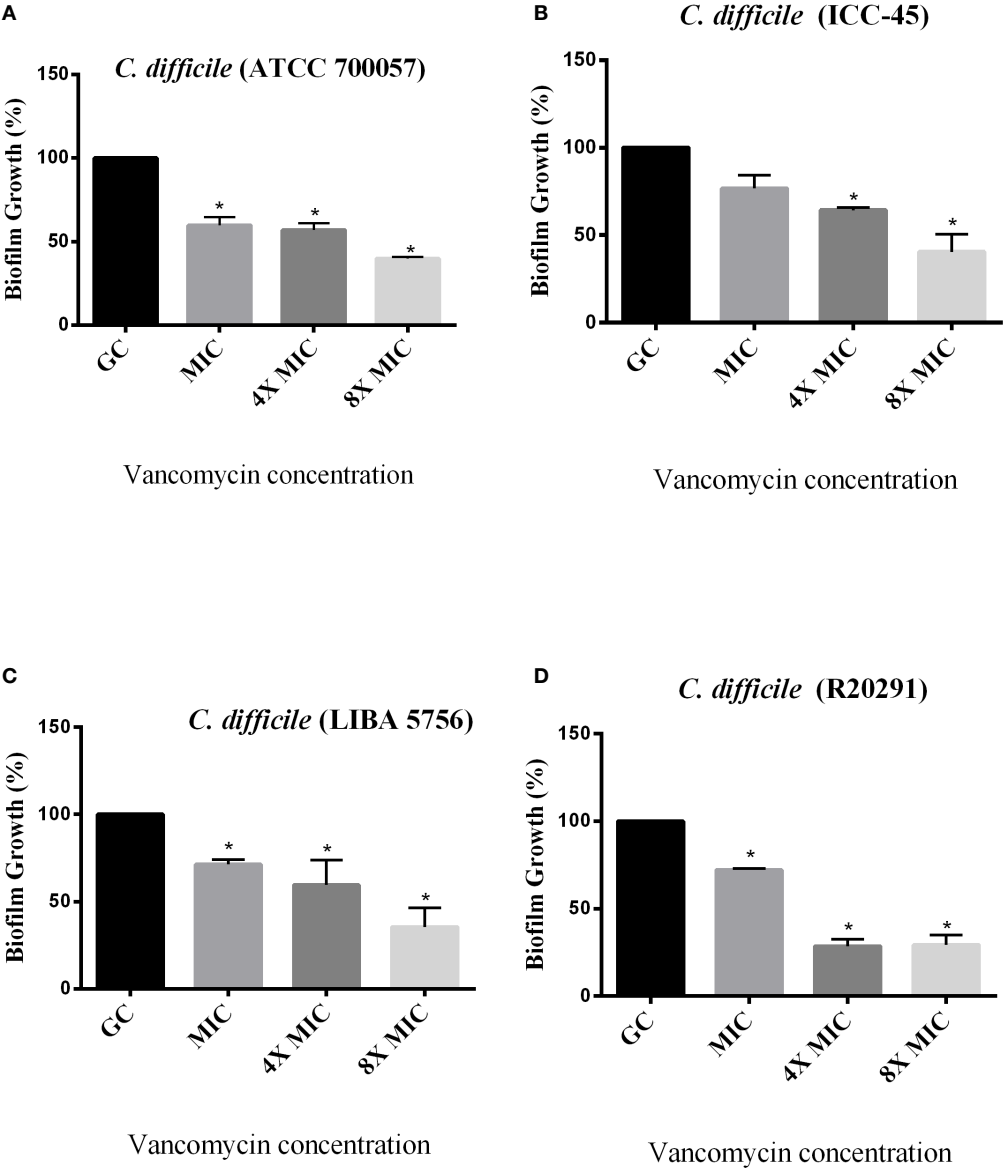
Effect of Vancomycin on growing biofilms of *C. difficile* strains ATCC 700057 **(A)** ICC-45 **(B)**, and epidemic strains LIBA 5756 **(C)**, R20291 **(D)**. *indicates statistically significant differences between the tested concentrations and growth control (GC). (One-way ANOVA followed by Tukey test).

**Figure 7:**
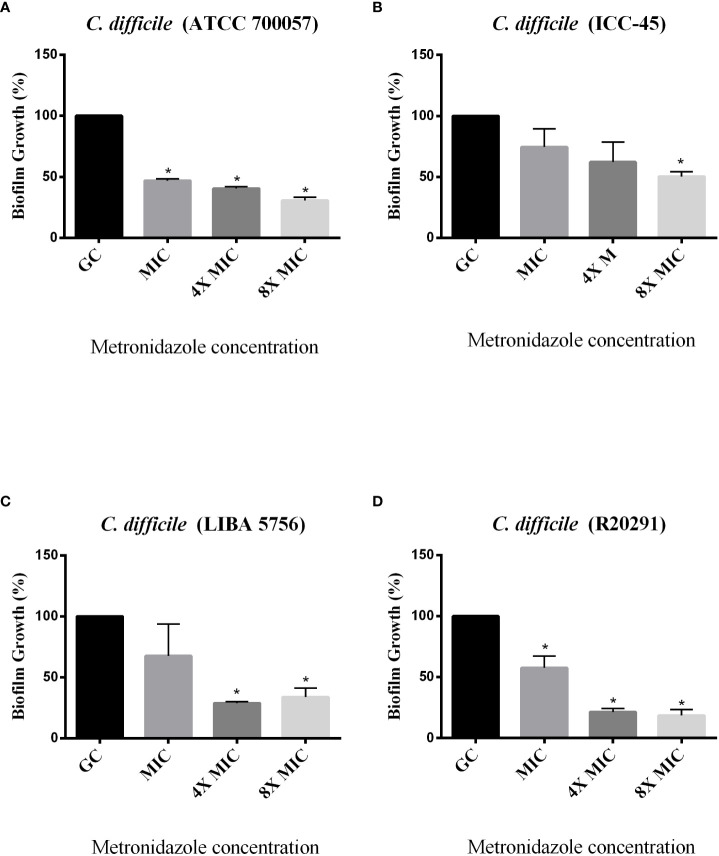
Effect of Metronidazole on growing biofilms of *C. difficile* strains ATCC 700057 **(A)** ICC-45 **(B)**, and epidemic strains LIBA 5756 **(C)**, R20291 **(D)**. *indicates statistically significant differences between the tested concentrations and growth control (GC) (P < 0.001). (One-way ANOVA followed by Tukey test).

The effect of antimicrobials against *C. difficile* strains was first determined in planktonic cells using the broth microdilution method. For all strains, the VAN MIC ranged from 0.5 to 2 μg/mL (**Table 3**) MTZ MICs were 2 for ATCC 700057, LIBA 5756 and R20291, while ICC-45 was 16 μg/mL. The effects of VAN or MTZ on *C. difficile* biofilm formation were investigated at MIC, 4xMIC, and 8xMIC concentrations. The effects of VAN or MTZ inhibited biofilm formation in epidemic strains in a dose-dependetn manner, resulting in a significant reduction in biofilm biomass (**Figures 6**, **7A–D**). Except for ICC-45 strain, MIC concentrations of VAN and MIC, as well as 4xMIC of MTZ, did not inhibit biofilm formation (**Figures 6B**, **7B**).

**Table 3:** Minimum inhibitory concentrations (MICs) of MITZ and VAN against Clostridioides difficile starins.

Strain	Metronidazole _(MIC 90%)_	Vancomycin _(MIC 90%)_
ATCC 700057	2 μg/mL	0.5 μg/mL
ICC-45	16 μg/mL	1.0 μg/mL
LIBA 5756	2 μg/mL	0.5 μg/mL
R20291	2 μg/mL	0.5 μg/mL

MIC 90%- minimum inhibitory concentration at 90%.

## 4 Discussion

We demonstrated that the three MLST Clade 2 strains tested (ICC-45, NAP1/027/ST01 (LIBA5756 and R20291), as well as the non-toxigenic strain ATCC700057, are capable of forming biofilms. The study of biofilms formed by a non-toxigenic strain contributes to determine the clinical significance of non-toxigenic *C. difficile* strains, which is controversial. According to the popular belief, it primarily belongs to the normal microbiota of the human intestinal tract. Several studies, however, have found non-toxigenic *C. difficile* in the stool of hospitalized patients with persistent diarrhea, implying that these strains may contain other virulence factors that can play a role in colonization and disease development (Predrag et al., 2012).

It is well established that microbial biofilm a critical virulence factor associated with the pathogenesis of *C. difficile* and its recurrent infections, as it contributes to the bacterium’s survival. The biofilm influences antimicrobial resistance and protects bacteria from environmental stress and the host’s immune response (Dapa and Unnikrishnan, 2013; Soavelomandroso et al., 2017). Thus, biofilm characterization improves understanding of strain behavior and can aid in the development of novel strategies to prevent or treat *C. difficile* infection.

Among the tested strains, the epidemic NAP1/027/ST01 (RT027) was linked to outbreaks and more severe infections around the world. During a 2010 outbreak in Costa Rica, the NAP1/027/ST01 (LIBA5756) was isolated. However, NAP1/027/ST1 has not been identified as a cause of outbreaks in Brazil. Costa et al. (2016), on the other hand, demonstrated that ICC-45 is phylogenetically related to NAP1/027, both of which are members of the MLST hypervirulent Clade 2. In the present study, all the strains, including the non-toxigenic strain ATCC 700057, were capable of producing moderate biofilms *in vitro* (1.1 <DO_570_>3.5), after 24, 48, 72 and 120h of incubation, according to Pantaléon et al. (2018) classification. Even though LIBA 5756 produced the most biofilm biomass after 48h, its production significantly decreased after 120h when compared to the biofilm production capacity of ICC45. Interestingly, even after 120h, the ICC-45 biofilm was very similar to the R20291 biofilm, which could contribute to its virulence and antibiotic resistance. These findings corroborates with previous research indicating biofilm formation is time and strain-dependent (Dapa and Unnikrishnan, 2013; Walter et al., 2015; Vuotto et al., 2018).

Biofilm formation has been associated with essential aspects of colonization and persistence *in vivo* (Pizarro-Guajardo et al., 2016). The extracellular matrix is essential for the maintenance of the biofilm, as it creates a stable microenvironment (Karygianni et al., 2020), which provides protection to the microorganisms contained within the biofilm (Yin et al., 2019). For that reason, we used SEM to examine the biofilm extracellular matrices and the embedded cells. The ATCC 700057 strain had a thinner biofilm after 72h, whereas the epidemic NAP1/027 strain had a dense biofilm with masses of agglomerated cells. Small ovoid structures in the ICC-45 biofilm were observed. Based on SEM observations made by Semenyuk et al. (2014) with another C. difficile strain, we hypothesize that these ovoid cells are spores. The existence of spores was further confirmed by the authors using phase contrast microscopy, an imaging technique that distinguishes these structures from vegetative cells. Similar findings were found in the biofilm of another strain of *C difficile*, and the authors used transmission electron microscopy to identify these ovoid cells as spores (Semenyuk et al, 2014). Spores are bacterial survival structures that can resist antibiotic therapy, contributing to disease recurrence and making it hard to treat *(* Frost et al., 2021). According to a study conducted by James et al. (2018), antibiotics such as MTZ and VAN are ineffective in reducing the amount of spores within a biofilm compared to other antibiotics. We observed in the present study that ICC-45 strain is more resistant to VAN or MTZ than epidemic strains. We should also consider that the antibiotic resistance observed in the ICC-45 biofilm can be attributed to the presence of specific extracellular matrix proteins, as we previously showed that ICC-45 produces and expresses more proteins related to antibiotic resistance of the beta-lactamases family, pyruvate-ferredoxin oxidoreductase and nitroreductase when compared to strain NAP1/027 (LIBA 5756) (de Melo Pacífico et al., 2021).

The sporulation of *C difficile* is associated to the *spo*0A gene of *B subitilis*, which is the master regulator of sporulation in all endospore-forming bacteria (DiCandia et al., 2022). In our study, the ICC-45 biofilm exhibited a higher *spo*0A gene expression in a 3-day-old biofilm. This result may indicate that ICC-45 sporulation process begins earlier for this strain, which may contribute to the virulence.

In the present study, we also investigated the *C. difficile* TcdA protein expression. According to Semenyuk et al. (2014), increased toxin levels in *C. difficile* biofilms occur after 3 days. TcdA levels from the matrix of 72h biofilms from LIBA 5756 and R20291 toxigenic strains were higher than ATCC 700057 (non-toxigenic) and ICC-45 biofilms. Our result is in agreement with a previous study that reported lower release of TcdA by the ICC-45 strain compared to NAP1/027 (Costa et al., 2016). However, another study of our group, using high‐performance liquid chromatography‐tandem mass spectrometry, demonstrated increased proportions of TcdA in the exoproteome of ICC-45 compared with NAP1/027 (de Melo Pacífico et al., 2021). This disparity is most likely due to changes in the cultivation techniques and periods across studies. Furthermore, unlike previous studies, we evaluated biofilms rather than isolated cells in culture. Interestingly, here, the 72h-old ICC-45 biofilm exhibited higher gene expression of *tcdA, tcdB, tcdC, slpA* and *spo*0A than the toxigenic R20291 and/or LIBA 5756 strains. Since mRNA is eventually translated into protein, this result may indicate a subsequent increase in *tcd*A gene production by the ICC-45 biofilm after 72h, but further studies are needed to confirm that hypothesis.

*C*. *difficile* adhesion to the *host colon epithelial* cells is *an* essential *stage* in the *pathophysiology* of *C*. *difficile* toxin-induced intestinal damageinjury. Therefore, we examined the gene expression of *C. difficile* cell surface-associated *proteins, such as* Cwp66 and Cwp84, both members of the Cell Wall Protein (Cwp) family. After 72h of incubation, all tested strains had equivalent *cwp*66 and *cwp*84 gene expression in their biofilms. The same result was found by Maldarelli et al. that reported *cwp*66 expression in a 1-week-old *C. difficile* biofilm (Maldarelli et al., 2016). The ICC-45 biofilm, on the other hand, had higher *slpA* gene expression than the biofilms from toxigenic strains LIBA 5756 and R20291. The *slp*A, is a single gene that encodes *surface layer* (*S-layer) proteins* (Bradshaw et al., 2018). S-layers allow bacteria to adhere to host cells or other environmental surfaces and protect cells from aggressions such as phagocytosis and harmful enzymes (Fagan and Fairweather, 2014). It has been described that S-layer is essential forproduction of *C. difficile* biofilm (Dapa and Unnikrishnan, 2013).

In conclusion, this study demonstrated that the three MLST Clade 2 evaluated are competent biofilm-forming bacteria, implying their ability to induce *C. difficile* infection recurrence, making treatment challenging. The greater expression of SlpA and Spo0A and antibiotic resistance observed in the ICC-45 biofilm may be associated with increased adhesion, sporulation and virulence. Our results draw attention to epidemiology surveillance focused on the emergence of resistant and recurrence-causing strains, in the face of a globalized world.

## Data availability statement

The original contributions presented in the study are included in the article/supplementary material. Further inquiries can be directed to the corresponding author.

## Author contributions

MM, wrote the manuscript and perfomed experiments, MS, helped in carrying out the experiments, CM, performed the statistical analysis. RL, wrote sections of the manuscript, DP, CQ-G, contributed to manuscript revision, DB, EF and GB contributed to conception and design of the study and wrote sections of the manuscript. All authors contributed to the article and approved the submitted version.

## Funding

GB is a recipient of a fellowship of the coordination of higher education personnel [CAPES]. This study was supported by PRONEX/ FUNCAP/CNPq of Brazil through Grant PR2-0101-00060.01.00/15 and (CNPq/Universal 2021/n.408779/2021-7) credited to GB.

## Acknowledgments

The authors thank Central analytical, Ceará Federal University, for logistical support.

## Conflict of interest

The authors declare that the research was conducted in the absence of any commercial or financial relationships that could be construed as a potential conflict of interest.

## Publisher’s note

All claims expressed in this article are solely those of the authors and do not necessarily represent those of their affiliated organizations, or those of the publisher, the editors and the reviewers. Any product that may be evaluated in this article, or claim that may be made by its manufacturer, is not guaranteed or endorsed by the publisher.
